# Mindful Sustainable Aging: Advancing a Comprehensive Approach to the Challenges and Opportunities of Old Age

**DOI:** 10.5964/ejop.v11i3.949

**Published:** 2015-08-20

**Authors:** Håkan Nilsson, Pia H. Bülow, Ali Kazemi

**Affiliations:** a School of Health and Welfare, Jönköping University, Jönköping, Sweden; b School of Health and Education, University of Skövde, Skövde, Sweden; c Department of Social Work, University of the Free State, Bloemfontein, South Africa; Aalborg University, Aalborg, Denmark

**Keywords:** mindfulness, social, existential, sustainable, ageing, activity theory, disengagement, successful aging, gerotranscendence

## Abstract

The primary aim of this article is to present a new concept called mindful sustainable aging (MSA), which is informed by mindfulness practices that support the physical, the mental, and especially, the social and the existential dimensions of old life. The concept of MSA is discussed and compared with four influential psychosocial theories in the field of gerontology, i.e., activity theory, disengagement theory, successful aging theory and gerotranscendence theory. The article ends with reviewing research on how mindfulness practice can help to manage, diminish and/or improve a number of serious physical conditions that are common among older people. The potential of mindfulness when it comes to facilitating for older adults in their quest for spiritual and existential meaning is discussed extensively throughout the article.

Each important phase of life carries its own set of psychological, existential and practical challenges, and joining the ranks of older persons is certainly no exception. Many among old people must cope with changing family roles and relationships as well as the reduction of financial resources, which limits the opportunity for recreation, travel, hobbies, personal purchases and so forth (Eliopoulos, 2014; Glicken, 2006). And yet, despite its many challenges and inconveniences, old age can be a highly productive, highly rewarding and ultimately meaningful period of life, depending on how one approaches, confronts and engages in this final transitional phase.

Within the human sciences the field of gerontology since the 1940s, has studied the psychological, social and biological dimensions of aging. Social gerontology is a sub-discipline of this field that over the years has developed a number of psychosocial theories of aging, each of which attempts to explain what it takes for people to maintain a high quality of life when becoming old without giving in to despair, depression and/or extreme inactivity. Four of the more common among these various theories are *activity* theory (Havighurst & Albrecht, 1953), *disengagement* theory (Cumming & Henry, 1961), *successful aging* theory (Rowe & Kahn, 1987), and *gerotranscendence* theory (Tornstam, 1996).

The primary aim of this article is to enter into the ongoing theoretical discussion on healthful aging by introducing a new concept – *mindful sustainable aging* (MSA) – a concept that encompasses all important gerontological dimensions, thus providing a more comprehensive approach to the facilitation of old age. MSA places mindfulness practice and thought in a pivotal role, introducing it as a beneficial, life-enhancing tool that promotes physical and mental fitness, encourages active social interaction, and focuses the older person’s mind on the search for existential meaning, self-discovery and peace in this final transitional phase (cf. Nilsson & Kazemi, 2015).

Toward this end, we begin by providing a brief overview of mindfulness practice as it relates to the physical and the mental, but most especially, the social and the existential dimensions of life. Subsequently, brief descriptions of the four aging theories mentioned above are provided. Then the concept of MSA is described and compared with the aging theories. Finally, we provide some examples of how mindfulness practice can help to manage, diminish and/or improve a number of serious physical conditions that are common among older adults, concluding with a description of the primary distinguishing features of MSA.

## A Multi-Dimensional View of Mindfulness

Mindfulness, as it applies to both the social and the existential dimensions of old age, represents a primary pillar of the comprehensive concept of MSA that will be presented herein, although the contribution of mindfulness to the physical and mental health of older adults is of significance as well. A brief description of all four dimensions is provided in the following (see Nilsson, 2014):

1. The *physical* dimension of mindfulness is focused on the body’s moment-by-moment state of being as well as on routine bodily activities. The latter defined as “any bodily movement produced by skeletal muscles that results in energy expenditure” (Caspersen, Powell, & Christenson, 1985, p. 126). Through the practice of body scanning, meditation and yoga older adults are able to heighten their awareness of the body’s various states, improve their overall physical conditioning, and take better notice of any health problems that might arise. Apart from this, mindfulness training provides the older person with an excellent opportunity (or excuse) to exercise the body in a regulated way.
2. The *mental* dimension of mindfulness, which also is addressed through yoga, body scanning and meditation, and can help in the regulation of various mental states, is of importance when it comes to the management of stress, anxiety and depression in old age. Mindfulness practice also serves to stimulate and rejuvenate one’s mental faculties.
3. The *social* dimension of mindfulness encourages social interaction and involvement by engaging the older person in group activities that are enjoyable and challenging. Moreover, one of the primary social goals of mindfulness training is to increase the groups’ feelings of empathy and compassion, not only in terms of the other members of the group, but also in terms of their own lives and the world at large. Such positive social feelings tend to enhance one’s sense of inner integrity and deepen one’s wisdom^i^ (Erikson, 1959/1980).
4. The *existential* dimension is approached herein from epigenetic (Erikson, 1997) and logotherapeutic (Frankl, 1978) perspectives. Although the term epigenetic originally springs from the field of biology, Erikson employed it in a very specific sense that relates to the stages of psychosocial development, noting that the unfolding of human personality progresses in predetermined stages that are influenced by the surrounding sociocultural milieu, with each stage of development being to some degree influenced by how the previous stages have been resolved. Logotherapy is based on Frankl’s realization that the primary driver in human life is not the pursuit of power and/or enjoyment, but rather *the search for meaning*. While the search for meaning is important regardless of one’s age, it is of crucial importance for those who are coming close to the terminus of life.

## Highlighting the Social and Existential Dimensions of Mindfulness

Sooner or later each of us must come to terms with the Buddhist triad of old age, disease and death. Becoming old marks the beginning of life’s longest developmental phase, and one that will entail the challenges of retirement and finding renewed (and eventually ultimate) meaning in life. The extent to which one experiences meaning in old age is related to the manner in which one has lived one’s life – the skills and competencies that one has accumulated, the caliber and quality of one’s interpersonal relationships, the degree to which one has developed kindness, empathy, and compassion.

Using Erikson’s developmental stage theory (Erikson, 1997) will inform our exploration of the ways in which mindfulness can enable the older person to successfully resolve what Erikson viewed as the final crisis of life: integrity vs. despair, with integrity being the quality of ego that should be developed in old age.

Here *integrity* is said to consist of the ability to look back on one’s life with a feeling of satisfaction, peace and gratitude for all that has been given and received. Erikson (1959/1980) notes in this regard:

The possessor of integrity is ready to defend the dignity of his own lifestyle against all physical and economic treats. For he knows that an individual life is the accidental coincidence of but one life cycle within but one segment of history; and that for him all human integrity stands and falls with the one style of integrity of which he partakes (Erikson, 1959/1980, p. 104).

Thus, persons derive a sense of meaning (i.e., integrity) through careful review of how their lives have been lived (Krause, 2012). Ideally, however, integrity does not stop here, but rather continues to evolve into the virtue of wisdom.

A “wellderly” person who has achieved wisdom can meet the terminus of life with open-mindedness, curiosity and acceptance (i.e., the positive outcomes of the psychosocial crisis). We can assume that for Erikson the wise and “wellderly” have been able to integrate all other positive outcomes during their journey – from hope (*Stage 1*) to care (*Stage 7*). This is in contrast to the “illderly” who develops a *despairing* attitude toward *being in life* and a *disgust* toward what has been and is about to come. The “illderly” in other words meets the end of life with horror, regret and resentment (i.e., the negative outcomes of the psychosocial stage crises) (Wykle, Whitehouse, & Morris, 2005). We turn now to the existential dimension and the search for meaning.

Frankl has defined logotherapy as “meaning centered (psycho) therapy” (Frankl, 1978, p. 19) – an approach to the search for ontological meaning in life. According to Frankl, the search for meaning is a vital driving force of human life since human beings are oriented towards meaning in all circumstances (Frankl, 1979). Referring to Frankl, Kimble and Ellor (2001) note that the “crisis of aging appears to be a crisis of meaning. The challenge of older adulthood is to make sense of life at a stage when changes and losses occur with bewildering and sometimes overwhelming frequency and intensity” (Kimble & Ellor, 2001, p. 14).

To those facing catastrophic illness in old age, Frankl’s (1988) perspective offers hope for meaning through *dereflection*^ii^: “Although the helpless victim of a hopeless situation, a man who meets a fate that he cannot change can raise himself above himself, can grow beyond himself and thereby transform himself. He can transform a personal tragedy into a triumph” (Frankl, 1988, pp. 153-154).

Frankl considered suffering to be an inevitable part of life: “/…/ life´s meaning is an unconditional one, for it even includes the potential meaning of suffering” (Frankl, 1979, p. 181). Not only surviving but also triumphing over the most horrendous of human circumstances, Frankl himself stands as a prime example, both embodying and epitomizing Nietzsche’s noteworthy maxim that “he who has a *why* to live for, can bear almost any *how”* (Peterson, 2000, p. 6). Mindfulness practice, as rooted in Buddhist thought, can help the older person to rediscover their own “*why”* to live for. In this regard, mindfulness and logotherapy share a common feature, i.e., *self-transcendence*, defined by Frankl (1975) as that aspect of human existence which “is always directed to something, or someone, other than itself—be it a meaning to fulfill or another human being to encounter lovingly” (Frankl, 1975, p. 78).

## Psychosocial Theories of Aging

In the field of gerontology, a myriad of theories of aging have been advanced. Relevant to the main purpose of the present article, we will briefly describe four well-known theories, i.e., activity theory, disengagement theory, successful aging theory and gerotranscendence theory. Although each of these theories posits its own idea about how the psychosocial problems and challenges of aging can be best handled, they all share the common aim of studying and working with older adults so as to bring greater ease and enrichment to their lives.

### Activity Theory

Developed by Havighurst and Albrecht in 1953, *activity theory* addresses the issue of how persons can best adjust to the changing circumstances of old age – e.g., retirement, illness, loss of friends and loved ones through death, etc. In addressing this issue they recommend that older adults involve themselves in voluntary and leisure organizations, child care and other forms of social interaction. Activity theory thus strongly eschews the notion of a sedentary lifestyle and considers it essential to health and happiness that the older person remains active physically and socially. In other words, the more active older adults are the more stable and positive is their self-conception, which in turn leads to greater life satisfaction and higher morale (Havighurst & Albrecht, 1953).

### Disengagement Theory

Disengagement theory, developed by Cumming and Henry in the 1950s, emphasizes in contrast to activity theory that older adults should not be discouraged from following their inclination towards solitude and greater inactivity. While not completely discounting the importance of exercise and social activity for the upkeep of physical health and personal wellbeing, disengagement theory is opposed to artificially keeping the older person so busy with external activities that they have no time for contemplation and reflection (Cumming & Henry, 1961). In other words, disengagement theory posits that older adults in all societies undergo a process of adjustment that involves leaving former public and professional roles and narrowing their social horizon to the smaller circle of family and friends. This process enables the older person to die more peacefully, without the stress and distractions that come with a more socially involved life.

### Successful Aging Theory

Developed by Rowe and Kahn in 1987, the notion of successful aging was formed as a reaction to another view conceiving older adults as societal burdens and the old age primarily characterized by illness and decline. Rowe and Kahn (1997) describe successful aging as involving “low probability of disease and disease-related disability, high cognitive and physical functional capacity and active engagement with life” (Rowe & Kahn, 1997, p. 433). Their view of successful aging is hierarchical, indicating that the three components are sequentially linked, with each having some degree of dependence on the one that comes before. It is also transitional in the sense that the older person can (and often do) move in and out of “success” at various points in their life. Finally, it is relative in the sense that its components are not intended to be descriptive of absolute states, and thus should not been used to classify the older person in terms of “success” and “failure”.

### Gerotranscendence Theory

Developed in 1996 by Tornstam, with input from theorists such as Jung and Erikson, gerotranscendence theory suggests that the very process of aging carries with it the potential to mature from a primarily materialistic, rational perception of the world to one that is more cosmic and transcendent – a transition that is normally accompanied by an increased level of satisfaction (Tornstam, 2011). Tornstam (2011) thus regards gerotranscendence as the final stage of a possible natural progression towards maturation and wisdom, which can involve a redefining of the one’s self and one’s relationship to others as well as a new existential outlook.

Gerotranscendence, however, does not call for withdrawal or disengagement from the world, as is sometimes erroneously believed – i.e., it is not a disguised version of the old disengagement theory. Rather it is a theory that focuses on inner reality and inner development, describing a developmental pattern that moves beyond the old dualism of activity vs. disengagement. Once again, its central argument is that satisfaction in old age can be maximized by shifting from an outward to an inward orientation, from a materialist-rational to a cosmic-transcendent perspective – a positive inner process that can enhance human wisdom and wellbeing and bears some resemblance to Erikson’s view of old age as discussed above.

## Mindful Sustainable Aging

While activity theory and successful aging theory both consider an active physical and social life to be most essential to healthful aging, disengagement theory and gerotranscendence theory both consider the withdrawn, contemplative and inward-directed life to be even more so. In these diametrical notions, we are confronted with an artificial and unnecessary dichotomy between activity and withdrawal, when it is obvious that for both diverse and overlapping reasons each is important in terms of meeting the challenges as well as the opportunities of later life. This is against this background that the concept of MSA is advanced.

**Figure 1:**
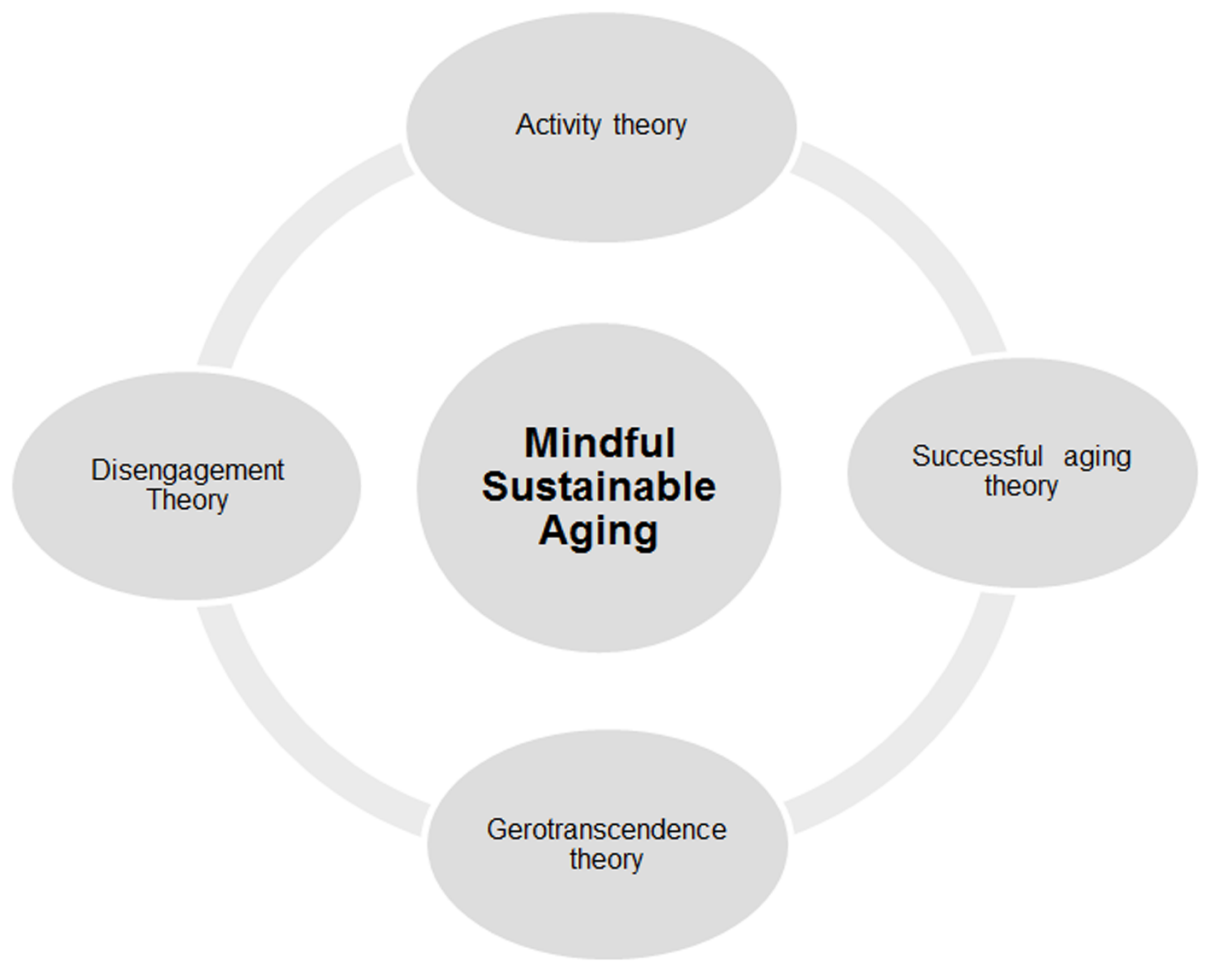
Mindful Sustainable Aging (MSA).

The concept of MSA does not represent a departure from these theories; rather, by adding the element of mindfulness to the notion of sustainability^iii^, it incorporates the crucial features of all four and attempts to go beyond them, at least in a few essential ways (see Figure 1). Physical and social activities as well as continued involvement in the world are certainly important for older adults. Equally important, on the other hand, are withdrawal and disengagement, which allow time for valuable contemplation.

Beyond these aspects, the concept of MSA, by drawing on Buddhist teachings (Atchley, 2003), specifically focuses on the spiritual/existential dimension of old age, that is, on better preparing the older person for the inevitable encounter with decline and death. By embracing the view that aging is rooted in inevitable loss, pain and death, the MSA concept constitutes a highly comprehensive and positive approach, seeing old age as an essential part of the human journey providing great opportunity for spiritual/existential growth and self-discovery. Echoing this view, Mowat (2005) notes that “The ageing individual is to discover and negotiate individual meaning even when confronted with what Frankl calls the tragic triad of pain, guilt and death. The task of old age and its fundamental purpose is therefore to search for meaning through a search for spiritual self. This is what… Erikson called ego integrity” (Mowat, 2005, p. 10).

MSA integrates the theories of activity, disengagement, successful aging, and gerotranscendence, and elaborates these by emphasizing mindfulness practice as a vital part of a sustainable healthful aging.

### MSA Encompasses the Need for Activity

Physical activity and exercise not only improve physical health and wellbeing of older adults (Mazzeo et al., 1998), but also provides the older person with regular purpose by bringing a certain amount of routine and social interaction to daily life. Regulated mindfulness practice, which includes stretching and yoga exercises, can be counted among the tools that offer such activity, which, in turn, tends to improve mental acuity, brighten one’s mood, add purpose to life, and enhance one’s ability to perform the common, ordinary actions – e.g., housework, gardening, athletics, travel and so forth (cf. Hicks & Connor, 2014).

Because some older adults lack the energy needed for everyday life they tend to become overly and unhealthily sedentary. Apart from the advantages mindfulness training offers during routine sessions, such practices also increase one’s energy level and thus help to foster a more active lifestyle (Alexander, Langer, Newman, Chandler, & Davies, 1989). It has been shown, for example, that older adults that regularly practice qi gong, tai-chi and other forms of mindfulness in parks and other outdoor venues, tend to be more healthy, resilient and active overall (e.g., Dean, 2001; Finger & Arnold, 2002).

Activity and stimulation in old age, however, are not only required for strengthening the body and increasing one’s energy level; it is also essential that the brain (or mind) remain active and stimulated as well. Regular mindfulness meditation and body-scanning provide stimulation for the brain, thus helping to make the older person more mentally fit (Brown, Ryan, & Creswell, 2007; Chiesa, Calati, & Serretti, 2011; McWilliams, 2014; Prakash, De Leon, Patterson, Schirda, & Janssen, 2014).

Finally, any discussion on the importance of activity in old age, must make some mention of sexuality, which need not remain the sole province of the young and middle aged. Recent research on mindfulness and sexuality indicates that regularly practicing yoga, body-scanning and meditation contributes positively to sexuality and can even help in the treatment of sexual dysfunctions (Kozlowski, 2013; Lazaridou & Kalogianni, 2013; McCarthy & Wald, 2013; Sommers, 2013). However, to our knowledge, there is a paucity of research specifically addressing mindfulness and sexuality among older adults.

### MSA Encompasses the Need for Disengagement

One way of characterizing activity theory and successful aging theory is as being more representative of the “doing” than the “being” mode, and the latter represented by the theories of disengagement and gerotranscendence.

Disengagement, what we would call entry into the being-mode, is highly facilitated by the sort of mindfulness training. Hick (2009) has noted in this connection that “as human beings we are more like ‘human doings’, keeping ourselves busy with endless activities and tasks. This often operates to distract us from our lives… This is where mindfulness comes in. It is nonjudgmental moment-to-moment awareness. In short, it is dwelling in the being mode with acceptance” (Hick, 2009, p. 7).

Mindfulness training and thought also enables the older person to further cultivate qualities like compassion, empathy and forgiveness not only toward others but also toward oneself (e.g., Nedeljkovic, Wirtz, & Ausfeld-Hafter, 2012). The quality of self-compassion is said to involve “being open to and moved by one’s own suffering, experiencing feelings of caring and kindness toward oneself, taking an understanding, nonjudgmental attitude toward one’s inadequacies and failures, and recognizing that one’s experience is part of the common human experience” (Neff, 2003, p. 224).

### MSA Fulfills the Requirements for Successful Aging

Mindfulness has been found to prevent cognitive decline (Gard, Hölzel, & Lazar, 2014) and to counteract stress (Moynihan et al., 2013) among older adults. Harmonious aging is further facilitated by practicing mindfulness (cf. Liang & Luo, 2012).

Freedom from disease and disability is one of the major ingredients of so-called successful aging, and it is in this connection that present-moment mindfulness training can help the older person to become more perceptive of the signs of physical and mental impairment. There are a number of disabling conditions that older adults are at risk of contracting – e.g., chronic pain, depression, heart disease, neurodegenerative disorders, urogenital problems, etc. How mindfulness can assist the older person in managing and improving these conditions, and thus help them to age successfully, will be more elaborately discussed later in this article.

Mindful practice positively affects optimism, resilience, effective coping styles and social and community involvement, and these qualities have been shown to be correlates of successful aging along with traditional measures of health and wellness (e.g., Montross et al., 2006).

Mowat (2005) discusses three perspectives on successful aging, i.e., the problem-based perspective, describing successful aging as the clever avoidance or overcoming of the vicissitudes of old age; the welcomed perspective, describing successful aging as living to the fullest and transcending the vicissitudes of old age, that is, rejecting the stereotypes of the problem-based model; and the spiritual perspective, describing successful aging as being able to negotiate and retain meaning through discovery of self. Echoing the third perspective, the concept of MSA views successful aging as a spiritual quest for meaning, maturation, fulfillment and wisdom (for a discussion of spirituality see Atchley, 2003; Zinnbauer et al., 1997).

### MSA Encompasses the Gerotranscendent Inward Turn

Old age is a time in which we have basically become free from much that has bound us to ordinary worldly life, where we can permit ourselves to live more according to our true character rather than the persona we have assumed so as to “get ahead” in life (Fromm, 1966). This phenomenon is akin to what Rosenmayr described as the “late freedom” of expanded consciousness in old age (Moody, 2003). Older adults are said to have a natural need for “alone time” – for thought and meditation, referred to as positive solitude. Solitude in this sense is meant to be understood, not in terms of disengagement, but rather as a transcendent state (Tornstam, 2011). This resonates with the Buddhist teachings that inform mindfulness practice (Bodhi, 2011).

Like gerotranscendence theory, MSA calls for a shift from the outward to the inward life, a shift that is facilitated by mindfulness practice, and which makes room for solitude, brings peace of mind and encourages the search for meaning and wisdom. In this regard Atchley (2003) noticed that “’Being while doing’ is a learned capacity that opens opportunities for the quality we call wisdom to enter into our world…Once this connection has been experienced, the experiencer is motivated to experience it again” (Atchley, 2003, p. 43).

## Mindfulness and Severe Physical Conditions

### Mindfulness and Chronic Pain

Many old persons experience pain in their everyday lives. Research on chronic pain patients has demonstrated that mindfulness training is an effective means of reducing the perception of pain intensity, emotional reactivity to pain, and the use of pain-relieving drugs, with benefits that have endured for up to four years later (Kabat-Zinn, 1982; Kabat-Zinn, Lipworth, & Burney, 1985; Kabat-Zinn, Lipworth, Burney, & Sellers, 1987).

### Mindfulness and Heart Disease

Coronary heart disease is responsible for the majority of heart attacks deaths in the United States (American Heart Association, 2001), as well as in Sweden (National Board of Health and Welfare, 2014). Most of the hearts attacks occur after the age of 65. With regard to regular mindfulness training, research findings indicate that it is an especially effective means of preventing or improving cardiovascular problems (Le Forge, 2005). In a study of women with heart disease, for example, Tacón, McComb, Caldera, and Randolph (2003) compared a treatment and a control group in terms of anxiety, emotional control, coping styles and health locus of control, with findings indicating superior results for those in the mindfulness-based stress reduction program. In another study, Alexander et al. (1989) compared transcendental meditation, mindfulness and relaxation training among older people, and found that participants in the meditation and mindfulness groups had lower blood pressure than those in the relaxation group three months after training; they also found that those in the meditation and mindfulness groups had better survival rates three years after the training.

### Mindfulness and Urogenital Problems

Urogenital disease is another common health problem, especially among older male adults for whom the prostate sooner or later becomes a problem. Living with prostate disease (e.g., prostatitis, prostatic hypertrophy and prostate cancer) and/or bladder disease (e.g., interstitial cystitis) is both a stressful and a painful experience. It has been found, however, that problems such as these can be alleviated with relaxation training – e.g., paradoxical relaxation (Wise & Anderson, 2010). Urologists argue that pelvic pain is connected to muscular tension in the levator ani (i.e., the floor of the pelvic cavity), and further note that stretching exercises as well as paradoxical relaxation (which is similar to mindfulness’s body scanning) can help to improve urogenital discomfort among both men and women. Although a few studies on the effects of mindfulness on urogenital problems among older adults have shown promising results (Carlson, Speca, Faris, & Patel, 2007; Chambers, Foley, Galt, Ferguson, & Clutton, 2012), more research needs to be conducted in this area.

### Mindfulness and Cancer

Cancer management is a major area in which mindfulness and other types of mind-body therapies have been found to be effective. Ott, Norris, and Bauer-Wu (2006), for instance, have noted that although the field is just beginning to grow, mindfulness-based interventions have shown remarkable benefits in terms of enhancing the wellbeing of persons living with cancer. Such forms of intervention are said to provide a healthy counterbalance to various negative experiences of most cancer patients.

## Mindfulness-Based Stress Reduction for Older Adults

Among the various forms of mindfulness programs that are currently available, *Mindfulness-based Stress Reduction* (or MBSR) is among the frequently used ones. MBSR was originally developed in 1990 by Kabat-Zinn (2004) for use among certain patients as a pain- and stress-reduction technique. As originally designed, MBSR consists of an eight-week training program for a group of no more than 30 persons, with each session lasting for approximately 2.5 hours. A training session involves the three standardized mindfulness practices of body scanning, sitting/walking (or wheelchair meditation for those with disabilities) meditation and *hatha yoga* (or gentle yoga for older adults with disabilities). Within that 8-week frame, one day is set aside as a retreat (Kabat-Zinn, 2004).

Participants in MBSR are advised to continue their practices outside the group by engaging in personal training for at least 45 minutes a day, six days a week. However, it is important to adapt the time and the training to the older adult’s capacity. Although audiotapes are available to assist in treatment, participants are encouraged to practice without such aids once their skills have been sufficiently developed. Whenever various emotions, sensations and/or cognitions arise during practice, participants are instructed to observe such occurrences with a nonjudgmental attitude (Baer, 2003).

While the reduction of pain and stress have been the original aim of the program, over the years MBSR has proved to be an efficient form of treatment for a wide range of maladies, from cancer to heart disease to depression to anxiety and also for older adults without significant frailty (Moss et al., 2015).

## Concluding Remarks

By drawing upon recent developments in the field of mindfulness emphasizing its multidimensional character, the concept of mindful sustainable aging was introduced and discussed. This notion represents an integrated approach for managing hazards of old age, i.e., physical illness, cognitive decline and death.

By engaging in things that demand both activity and spirituality, older people cultivate a mindful sense that is still vibrant with hope and meaning. However, older people also need time to cultivate their inner being by withdrawal from the outer realm of the doing-mode. Older adults who have made the shift from a doing-mode to a being-mode are considered to be spiritual elders. Generativity and wisdom are hallmarks for this group of older adults (Atchley, 2003). Mindfulness involves the practice and development of skills that facilitate and cultivate generativity and wisdom.

This notwithstanding, the mindfulness movement has managed to attract a great deal of interest among professional psychological and medical therapists as well as certified life coach instructors for managing maladies as stress, depression, anxiety, negative thoughts and feelings, severe physical discomfort and disorders. It is within the realm of this therapeutic framework that mindfulness training has proved beneficial in terms of bettering the lot of many individuals. Ultimately, MSA is about bettering the lives of older adults and enabling them to make the most of what their lives have to offer.

To sum up, learning to use mindfulness later in life can be important in terms of coping with the (psychosocial) crises of old age and the struggle to find meaning in late life. The fact that mindfulness spans both the normal and the pathological makes it capable of addressing a wide range of problems, something that this article has attempted to point out.
